# HoloLens augmented reality system for transperineal free-hand prostate procedures

**DOI:** 10.1117/1.JMI.10.2.025001

**Published:** 2023-03-01

**Authors:** Ming Li, Sherif Mehralivand, Sheng Xu, Nicole Varble, Ivane Bakhutashvili, Sandeep Gurram, Peter A. Pinto, Peter L. Choyke, Bradford J. Wood, Baris Turkbey

**Affiliations:** a National Institutes of Health, Center for Interventional Oncology, Radiology and Imaging Sciences, Clinical Center, Bethesda, Maryland, United States; b National Institutes of Health, Molecular Imaging Branch, National Cancer Institute, Bethesda, Maryland, United States; c Philips Research of North America, Cambridge, Massachusetts, United States; d National Institutes of Health, Urologic Oncology Branch, National Cancer Institute, Bethesda, Maryland, United States

**Keywords:** augmented reality, interventional radiology, percutaneous procedure, transperineal prostate procedure, HoloLens guidance, medical imaging

## Abstract

**Purpose:**

An augmented reality (AR) system was developed to facilitate free-hand real-time needle guidance for transperineal prostate (TP) procedures and to overcome the limitations of a traditional guidance grid.

**Approach:**

The HoloLens AR system enables the superimposition of annotated anatomy derived from preprocedural volumetric images onto a patient and addresses the most challenging part of free-hand TP procedures by providing real-time needle tip localization and needle depth visualization during insertion. The AR system accuracy, or the image overlay accuracy (n=56), and needle targeting accuracy (n=24) were evaluated within a 3D-printed phantom. Three operators each used a planned-path guidance method (n=4) and free-hand guidance (n=4) to guide needles into targets in a gel phantom. Placement error was recorded. The feasibility of the system was further evaluated by delivering soft tissue markers into tumors of an anthropomorphic pelvic phantom via the perineum.

**Results:**

The image overlay error was 1.29±0.57  mm, and needle targeting error was 2.13±0.52  mm. The planned-path guidance placements showed similar error compared to the free-hand guidance (4.14±1.08  mm versus 4.20±1.08  mm, p=0.90). The markers were successfully implanted either into or in close proximity to the target lesion.

**Conclusions:**

The HoloLens AR system can provide accurate needle guidance for TP interventions. AR support for free-hand lesion targeting is feasible and may provide more flexibility than grid-based methods, due to the real-time 3D and immersive experience during free-hand TP procedures.

## 1 Introduction

Prostate cancer is the second leading cause of cancer death among American men.^1^ The two principal approaches for the diagnosis of prostate cancer are transrectal and transperineal (TP) biopsy.^2^ Similarities in efficacy between the two biopsy approaches have been reported.^3^ However, the TP approach results in a markedly lower risk of sepsis.^4^

Same as prostate biopsy, many prostate cancer treatments can also be conducted via TP approach, such as focal laser ablation (FLA) and brachytherapy. FLA, a therapeutic treatment for patients with low to intermediate risk prostate cancer,^5^ uses high-energy laser that induces cell death through rapid heating at the targeted region. In a transperineal approach, the laser fibers are inserted into the target lesion via an introducer needle through the perineum wall.^6^ In prostate brachytherapy, radioactive seeds are placed into the prostate gland to deliver the radiation that can kill the cancer cells. The implantations of these radioactive seeds are typically performed via a transperineal approach.^7^

Magnetic resonance imaging (MRI) produces high-resolution images of the prostate, which is important for prostate cancer diagnosis and can be used to guide the placement of the needle.^8^ For in-gantry MRI-guided TP prostate procedure, such as biopsy, ablation procedures, or brachytherapy, a grid template with embedded MR visible fiducial markers is typically used to guide the needle or probe. However, this guidance method is limited to a parallel insertion trajectory with fixed spacing interval (e.g., 5 mm). The parallel insertion trajectory could limit access to lateral or anterior lesions located close to the edge of larger prostates because the pubic bone may block the straight path to the target and the fixed intervals may prevent the accurate biopsy of smaller targets.

MRI-ultrasound (US) fusion guided TP-procedure references the MRI targets via a transrectal US (TRUS) fused to the preprocedural MRI. Image-guided TP procedure is commonly achieved with a grid or with a coaxial free-hand approach. Both approaches use TRUS for registration and guidance. There is a novel approach recently described that uses free-hand transperineal US (TPUS) with free-hand needles.^9^ In this completely free-hand setting outside of the rectum, visualization of the needle can be challenging compared to TRUS, due to reduced conspicuity or out-of-plane trajectories. Augmented reality (AR) may address this disadvantage or limitation of this completely outside of the rectum approach. Recently, Bettati et al.^10^ demonstrated the feasibility of combining multimodality images into an AR system for potential applications in prostate biopsy.

Robot-assisted needle guidance may have some advantages over the grid template, such as limitless positioning resolution, and autonomous needle targeting and angulation.^11^^–^^13^ However, this technology has not been widely adopted or practiced due to a variety of limitations, including cost, ergonomics, and added procedural time and workflow complexity.

AR describes a technologically enhanced version of reality that superimposes digital information onto the real world via registration and an external display device. AR technology has been introduced into minimally invasive surgery and interventional radiology by superimposing computer-generated images and data from MRI and computed tomography (CT) scans onto the surface of patients.^14^ Through stationary, handheld mobile devices, or a head-mounted goggle device, the physician can “see” anatomy beneath the skin of the patient. AR guidance technology can also facilitate image-guided percutaneous interventions involving needle placements^15^^,^^16^ by overlaying a projected entry point and needle pathway directly onto a patient. Compared to traditional image guidance on a peripheral 2D monitor, AR enables the physician to optimize the needle path or treatment plan at the bedside. The recent development of the head-mounted smart-glasses further increases mobility by anchoring digital three-dimensional (3D) objects in physical space rather than overlaying them on a 2D display.^17^ Moreover, the head-mounted devices free both the physician’s hands while performing procedural tasks.^18^

A novel AR system is proposed using HoloLens to facilitate free-hand real-time needle guidance during TP procedures. This paper describes the AR system and reports the preliminary results of using HoloLens AR guidance in phantom studies.

## 2 Methods

**Fig. 1:**
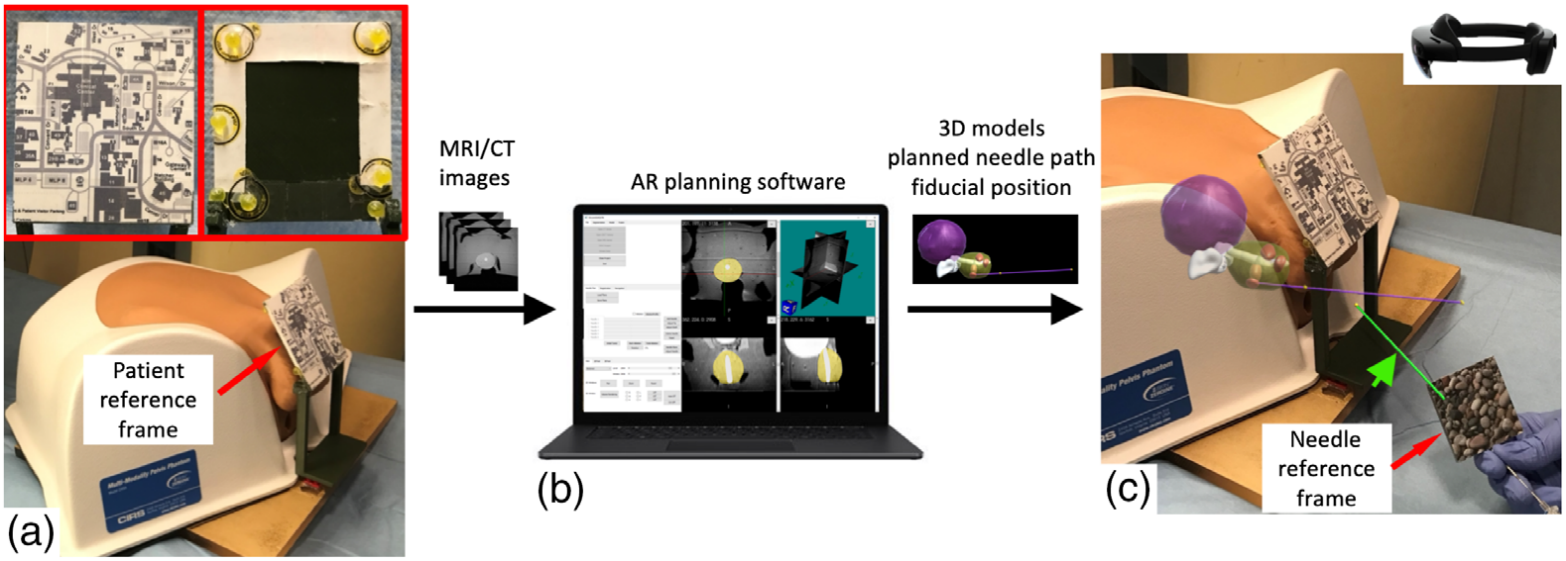
The HoloLens AR system components and workflow. (a) A CIRS 048A multimodality pelvis phantom with patient reference frame attached on the MRI/CT table. Insertion: close up view of front and back of the patient reference frame. (b) A DICOM volume was sent to the AR planning software for segmentation of anatomic features, interventional planning, and transformation between the reference frame and MRI/CT coordinates. (c) The anatomic features and interventional planning (e.g., needle path) uploaded and displayed on the HoloLens relative to the patient reference frame. Once the needle reference frame was tracked by HoloLens, the digital needle (identified by green arrow) was overlaid on the actual needle.

The AR system used volumetric 3D imaging data (such as MRI and/or CT) of the patient and was composed of a patient reference frame, a needle with or without a trackable needle reference frame attached, AR planning software, a novel AR application, and a HoloLens 2 (Microsoft, Redmond, WA). AR planning software was used for image segmentation, 3D model generation, and annotation of targets, as well as determining skin entry points with respect to preprocedural volumetric images. The system diagram of the AR system was shown in Fig. 1.

### 2.1 Transformation between Volumetric Imaging and HoloLens

The AR application superimposes the anatomic features of the preprocedure MRI and surgical plan onto the patient via a custom-designed patient reference frame (Fig. 1) that affixes to the patient. The width of the lower part of the plastic frame was the same as that of a grid template, therefore, the frame can be placed intimate with the perineum. There is a 95×70  mm working access window at the lower part of the frame. The upper part of the frame forms a 60-deg angle with the lower part and consists of a unique image with no repetitive patterns (90×80  mm). The 60-deg angle offers a comfortable viewing angle for the operator when the device camera is tracking the image pattern on the frame as well as the access window. Seven 6-mm diameter fiducial markers (Beekley Inc., Bristol, CT) were affixed at the back of the upper part of the frame [Fig. 1(a) insert]. The relative positions of the fiducials in the patient reference frame were measured with calipers.

The transformation between the HoloLens and preprocedural 3D MRI images was computed in two steps. In the planning stage, the seven fiducial markers were localized on the preprocedural MRI images, and the transformation from the image reference frame to the MRI frame, $T_{\mathrm{MRI}}^{\text{ImageFrame}}$, was computed by point-to-point registration on these fiducial markers. As the camera of the HoloLens identified and tracked images on the reference frame intraprocedurally, the transformation from the patient reference frame to the HoloLens, $T_{\text{ImageFrame}}^{\text{HoloLens}}$, was updated in real-time. The total transformation from the preprocedural MRI images to the HoloLens, $T_{\text{ImageFrame}}^{\text{HoloLens}}$, was computed as

$$T_{\mathrm{MRI}}^{\text{HoloLens}}=T_{\mathrm{MRI}}^{\text{ImageFrame}}\times T_{\text{ImageFrame}}^{\text{HoloLens}}.$$

### 2.2 Needle Tracking

To track the needle, a needle reference frame was attached to the biopsy needle [Fig. 1(c)]. The 50×50  mm needle reference frame was a 3 mm plastic flat plate, consisting of another unique image with no repetitive patterns. The angle between the plate and needle was 45 deg to allow for a comfortable viewing angle for the operator. The position of the needle with respect to the needle reference frame was predetermined and measured. As the camera of the HoloLens identified and tracked the needle reference frame, the position of the needle with respect to the AR device, $T_{\text{Needle}}^{\text{HoloLens}}$, was updated in real-time.

### 2.3 AR Application

**Fig. 2:**
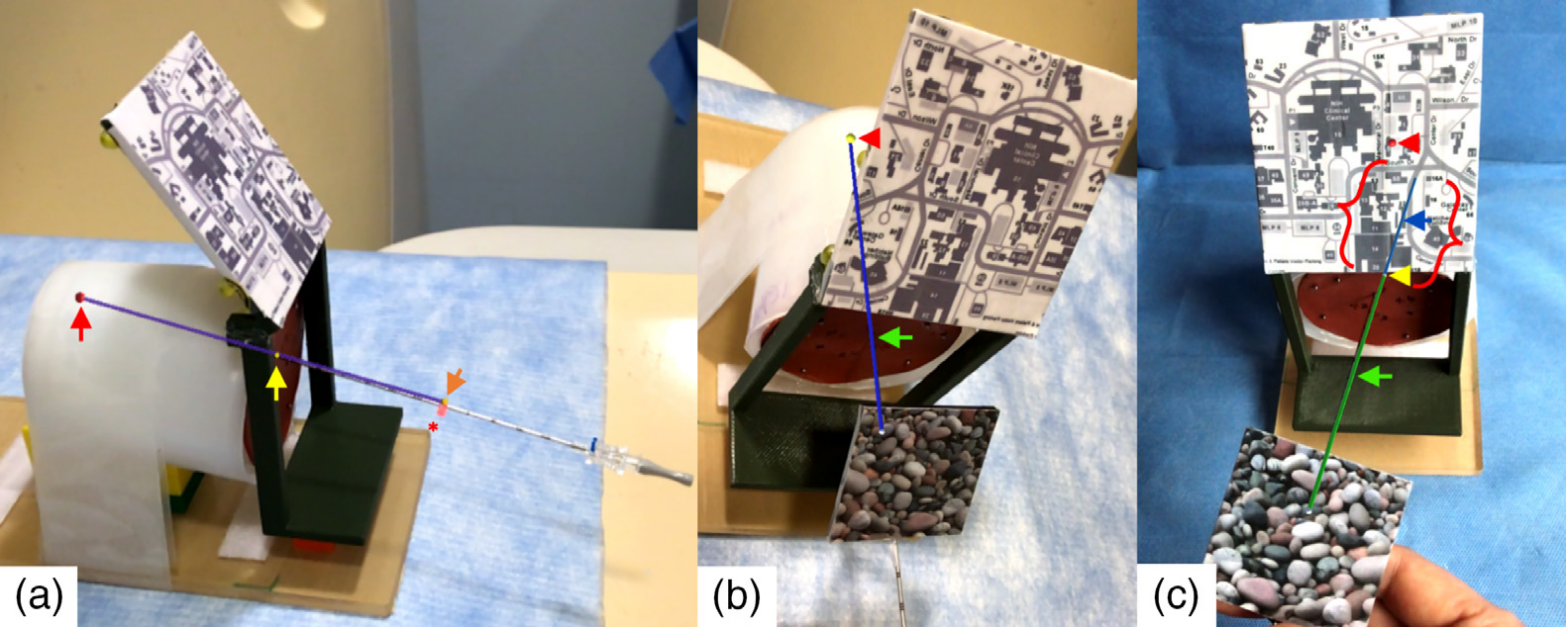
HoloLens AR features and guidance on a phantom used to evaluate the performance of HoloLens AR guidance in needle placement task. (a) The AR planned-path guidance superimposed the planned needle path (purple line), target (identified by the red arrow), entry point (identified by the yellow arrow), and planned needle length marker (identified by the orange arrow) on the phantom. The snapshot shows the alignment of the inserted needle with digital planned needle path. The actual needle length marker is identified by the red *. (b) and (c) AR free-hand guidance superimposed the digital target (identified by red arrowhead) on the phantom and digital needle (identified by green arrow) on the actual needle simultaneously. (b) The yellow digital target indicates the needle tip is within 5 mm vicinity of target, blue digital needle shaft indicates the correct needle angle. (c) Green digital needle indicates off-center needle insertion angle. Needle extension line (identified by red {}) points needle forward direction. These features help to assist the user to align the needle direction toward the target.

The AR application was developed using Unity 2019.4 (Unity Technologies, San Francisco, CA), Vuforia SDK 9.7 (PTC, Boston, MA), and MRTK 2.5 (Microsoft, Redmond, WA). In addition to the patient anatomy, the predefined treatment plan could be continuously superimposed onto the environment through the device display. Following selection of a planned needle trajectory, the planned target (red dot), needle path (purple line), skin entry point (yellow dot), and planned needle length marker (orange dot) were codisplayed [Fig. 2(a)].

Multiple assistance features were provided for monitoring the digital needle during insertion [Figs. 2(b) and 2(c)]. This included (1) an optional digital needle extension for casting the needle path, (2) digital needle shaft color changes to indicate proper needle angle, and (3) digital target color changes for indicating proximity of the needle tip to the target.

The AR application was operated by touch-less hand gestures and voice commands that let the user select the target from a preloaded target list, view the needle plan, view a 3D model of the surrounding anatomy, and turn on or off assistance features.

### 2.4 AR Guided Needle-Based TP Procedure: Planned-Path versus Free-Hand

The AR application provided two guidance methods (1) planned-path guidance and (2) free-hand guidance. Prior to the experiments, both guidance methods were proposed to eight interventional radiologists and urologists, and the preference rate was 50:50. Without loss of generality, both methods were implemented and evaluated. However, they were not mutually exclusive.

With the planned-path guidance method, the digital target lesion, skin entry point, and planned needle trajectory were displayed on the HoloLens and overlaid on the patient/phantom, whereas the actual needle was not tracked. The user used the displayed needle trajectory to align the actual needle, advance and adjust the actual needle through real-time comparison with the displayed needle plan and stop the insertion when the actual needle length mark overlapped with the planned needle length [Fig. 2(a)]. The user could move their head and view the patient from different angles as long as the patient image reference frame was kept in the camera’s field of view.

With the free-hand guidance method, only the digital target lesion was overlaid on the patient/phantom, no planned needle path was displayed. But the needle reference frame was tracked and the digital needle was displayed and overlaid on the actual needle. The user could choose any skin entry point. The user manipulated the needle and continuously verified the progress of the needle insertion through real-time digital feedback alongside assistance features provided by the AR application [Figs. 2(b) and 2(c)]. Both patient image reference frame and needle image frame had to be kept in the camera’s line of sight and field of view.

### 2.5 Experimental Setup

Prior to the MRI image guidance, the AR system accuracy was first evaluated using CT, because the metal fiducial was used to serve as ground truth for report error. Planning and verification CT images were acquired with a 16-slice clinical CT scanner (Brilliance CT; Philips, Cleveland, OH) using 2.0 mm-thick slices and 1.0 mm overlap. A 17GA ETW × 20 cm sterile placement needle (CIVCO Medical Solutions, Kalona, IA) was used.

For statistical analyses in the experiments, a Shapiro–Wilk test was first applied to assess whether the data were normally distributed. If the data were normally distributed, a Student’s t-test or one-way ANOVA was used to determine the statistical differences in results. Otherwise, a Mann–Whitney U test was used.

#### 2.5.1 Experiment 1: software and system accuracy

**Fig. 3:**
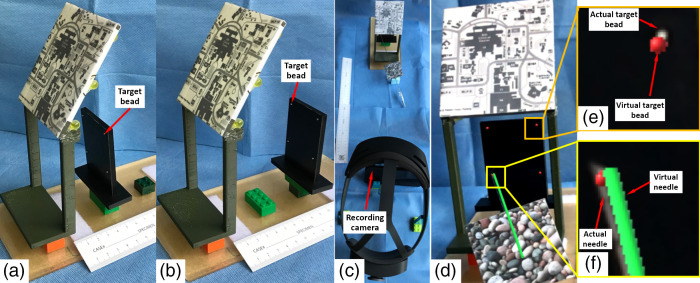
Experimental setup for measurement of the system accuracy. The target image overlay accuracy and visual needle targeting accuracy were measured. The plate with four metallic beads embedded was placed at (a) 5 cm and (b) 10 cm from the base of the image reference frame. (c) The experimental setup for image acquisition with HoloLens. (d) Image overlay and targeting overlay are displayed by the HoloLens, with the close-up views shown in (e) and (f).

The goal of the first experiment was to study the HoloLens AR system errors, without subjective human involvement. A plastic plate with four metallic beads embedded at the corner of a 40×40  mm square was used (Fig. 3). The plastic plate was placed such that the geometric center of four beads was located at the center of the working window of the patient reference frame. The plate was placed at two locations from the base of the patient reference frame, 50 and 100 mm, respectively. For each plate location, a CT scan was acquired. The beads were segmented from the CT images and the segmentation was uploaded onto the AR application and superimposed onto the actual plastic plate through the HoloLens displays.

Ideally, the system accuracy would be evaluated by directly comparing the 3D target display output of HoloLens and the actual target in the 3D space. However, measuring the 3D display output required stereoscopic vision, which is currently not possible. Instead, we studied the system accuracy by measuring the discrepancy between the actual target and the digital target that is codisplayed on one of the HoloLens’ lenses.

The registration error from CT to HoloLens via patient reference frame was evaluated as image overlay error view through HoloLens. The HoloLens was placed such that its own camera was faced and 45 cm from the patient reference frame [Fig. 3(c)]. A recording camera was placed at the calibrated optimal ocular position of the left lens to mimic the operator’s left eye. The phantom was placed at nine different positions with respect to the HoloLens and at each position a snapshot was acquired by the recording camera. Both actual beads and virtual beads were codisplayed on the 2D snapshot [Figs. 3(d) and 3(e)]. Image overlay error, which was defined as the distance (mm) from the center of actual beads to virtual beads on the 2D snapshot, was measured using ImageJ (NIH, Bethesda, MD).

The other AR system error, named visual needle targeting error, was also evaluated specifically for AR free-hand guidance, with which both the patient/phantom and needle need to be tracked. In addition to the above setup, an actual needle with the needle reference frame attached was placed in the field of view of HoloLens’ camera. The actual needle was placed such that the tip was touching each of the beads (n=8) from three different poses (n=3). Both the virtual bead and virtual needle were codisplayed on the 2D snapshot acquired by the recording camera placed at the calibrated optimal ocular position of the left lens of HoloLens. The visual needle targeting error, which was defined as the distance (mm) from the tip of the virtual needle to the center of the virtual bead, was measured using ImageJ.

#### 2.5.2 Experiment 2: performance of HoloLens AR guidance in needle placement task

**Fig. 4:**
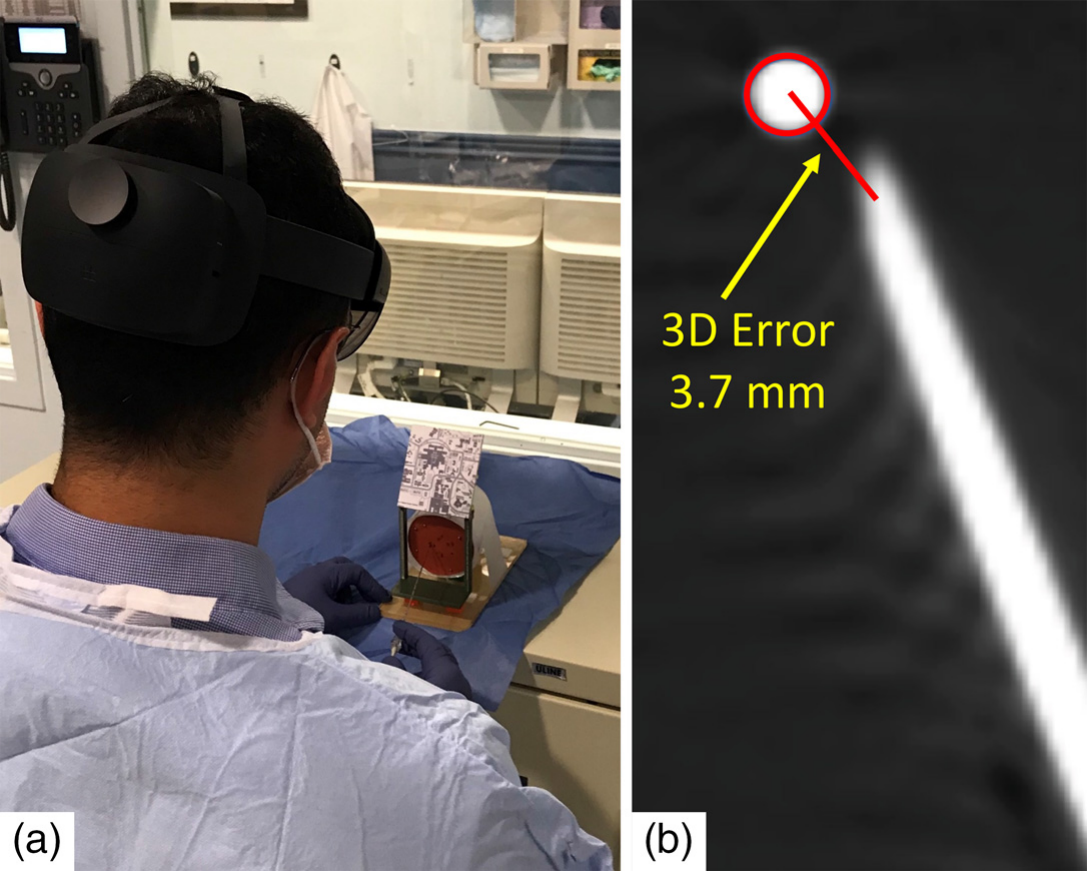
Experiment 2 – performance of HoloLens AR guidance in needle placement task. (a) Scene of a user executing a needle placement task and (b) measurement of the needle placement error was conducted on a postoperative CT. Needle placement error was defined as the distance from the needle tip to the center of the target bead.

The second experiment was to evaluate the performance of HoloLens AR guidance (Fig. 4). The phantom used in this study was constructed with acrylamide-based gel, representative of the mass density of human tissue,^19^ inside a cylindrical container (D=100  mm, L=120  mm). There were four metallic beads (D=1  mm) embedded inside the phantom at ∼6 to 8 cm in depth. A 1/16-inch flexible silicon sheet was placed on the exposed surface of the phantom to provide stability to the needle during insertions. The lower part of the patient reference frame was attached to the surface of the phantom, both the reference frame and phantom were secured on a base (Fig. 2). Needle entry points (n=4) were selected at random locations on the surface to include a variety of insertion depths (mean 79.6±3.1  mm) and angles (mean 12.8 deg ±  6.8 deg). Four 2 mm metal spheres were first placed on the phantom surface to identify needle entry points on preprocedural CT imaging.

Three operators (two experienced urologists and one nonphysician) were instructed to insert a total of eight needles into the phantom using the AR guided planned-path (n=4), or free-hand (n=4) for needle navigation (Fig. 4). For the AR guided planned-path method, all planned needle trajectories were generated and uploaded for subsequent display onto the HoloLens. For the AR-guided free-hand method, the virtual target and virtual needle were simultaneously displayed onto the HoloLens. Each operator had a training period of 20 to 30 min for practice prior to the experiment. The order of target and guidance methods were randomized between operators to control for possible learning and order effects. The same paired targets and entry points (but not sequence) were used for all operators with both methods. After each insertion, the inserted needle tips were compared with the target on post-CT scans. The needle placement time, defined as the time elapsed from the moment of needle puncture to navigation completion, was also recorded.

#### 2.5.3 Experiment 3: feasibility of HoloLens guidance in free-hand TP seed implantation procedure

**Fig. 5:**
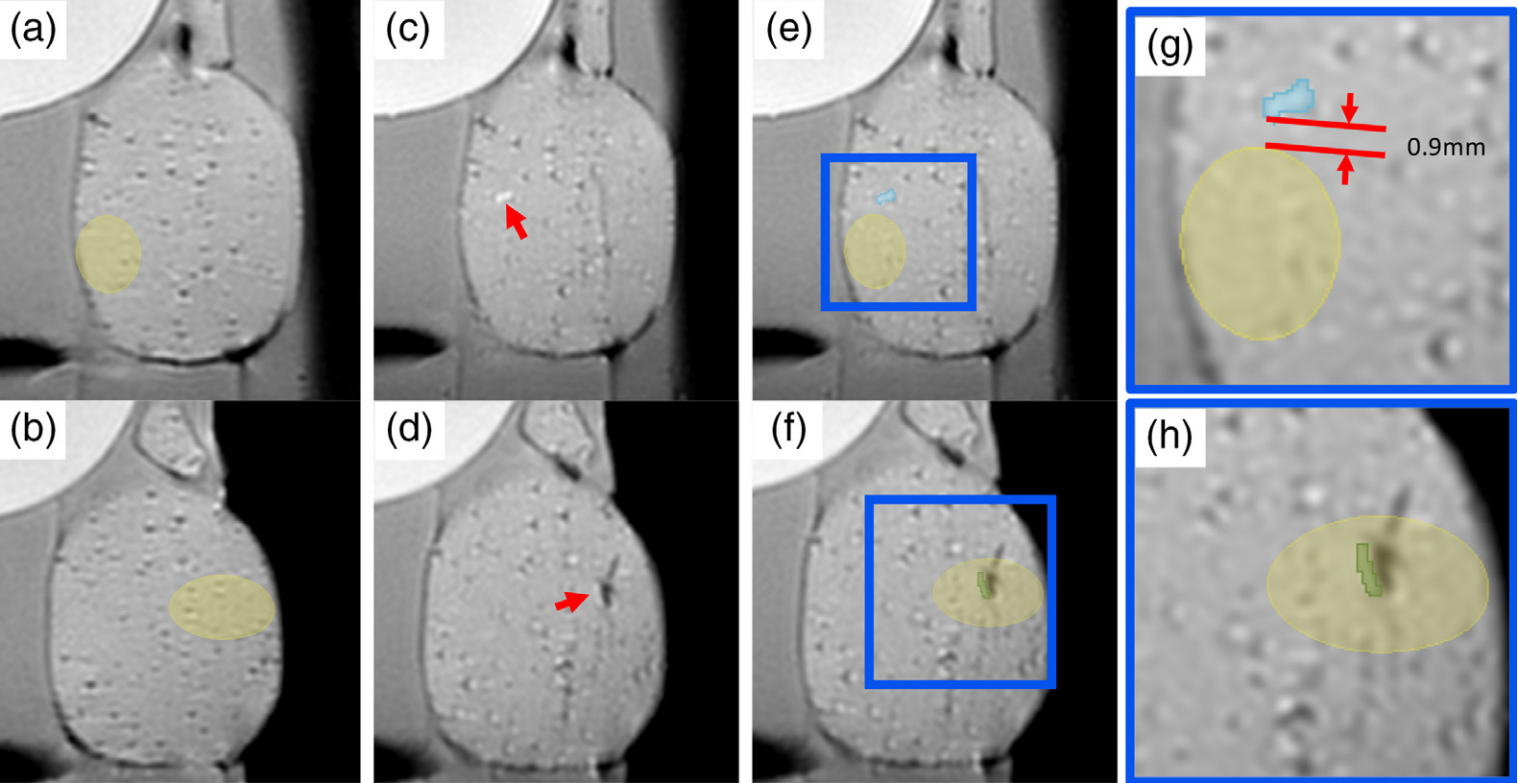
MRI images of seed implantation on two pseudotarget lesions in the pelvic phantom feasibility experiment. (a) and (b) Pre-op MRI with pseudotarget lesions marked. (c) and (d) Postinterventional MRI, seeds are identified by red arrow. (e) and (f) Pseudotarget lesions transformed to postinterventional MRI, with the seeds segmented. The highlighted area is shown in (g) and (h).

The third experiment was to evaluate the feasibility of the HoloLens AR guidance in free-hand TP procedure with an anthropomorphic phantom. A prostate and pelvic phantom (CIRS Model 048A, Norfolk, VA) containing a prostate, seminal vesicles, bladder, and rectum was scanned in MRI and anatomic structures were segmented for rendering a 3D model. Both seminal vesicles and pseudolesions served as lesion targets. Pseudolesions (0.5 to $0.7\;{\mathrm{cm}}^{3}$) representing prostate cancer were artificially added to the preprocedural MRI images [Figs. 5(a) and 5(b)]. The 3D models of artificial tumors, seminal vesicles, prostate, urethra, and bladder were displayed on the HoloLens and overlaid on the pelvic phantom [Fig. 1(c)].

Five 1.2×3  mm soft tissue gold markers were placed within the prostate and pelvic phantom using aforementioned 17GA ETW sterile placement needle under AR guidance by an experienced urologist. Both guidance methods were provided to help the operator plan the angle of attack prior to puncture and to monitor the progress of trajectory. After the markers were implanted, postinterventional MRI was scanned [Figs. 5(c) and 5(d)]. The pseudo lesions were mapped onto the post MRI based on the patient reference frame [Figs. 5(e) and 5(f)]. The distance between implanted soft tissue markers and the lesions was confirmed on the post MRI.

## 3 Results

Fiducial registration error between the CT coordinate system and the patient reference frame, which was defined as the root mean square (RMS) of the difference between the actual and calculated positions of the seven fiducials based on their image space positions and the registration matrix, was 0.81±0.34  mm from five scans.

### 3.1 Experiment 1: Software and System Accuracy

**Fig. 6:**
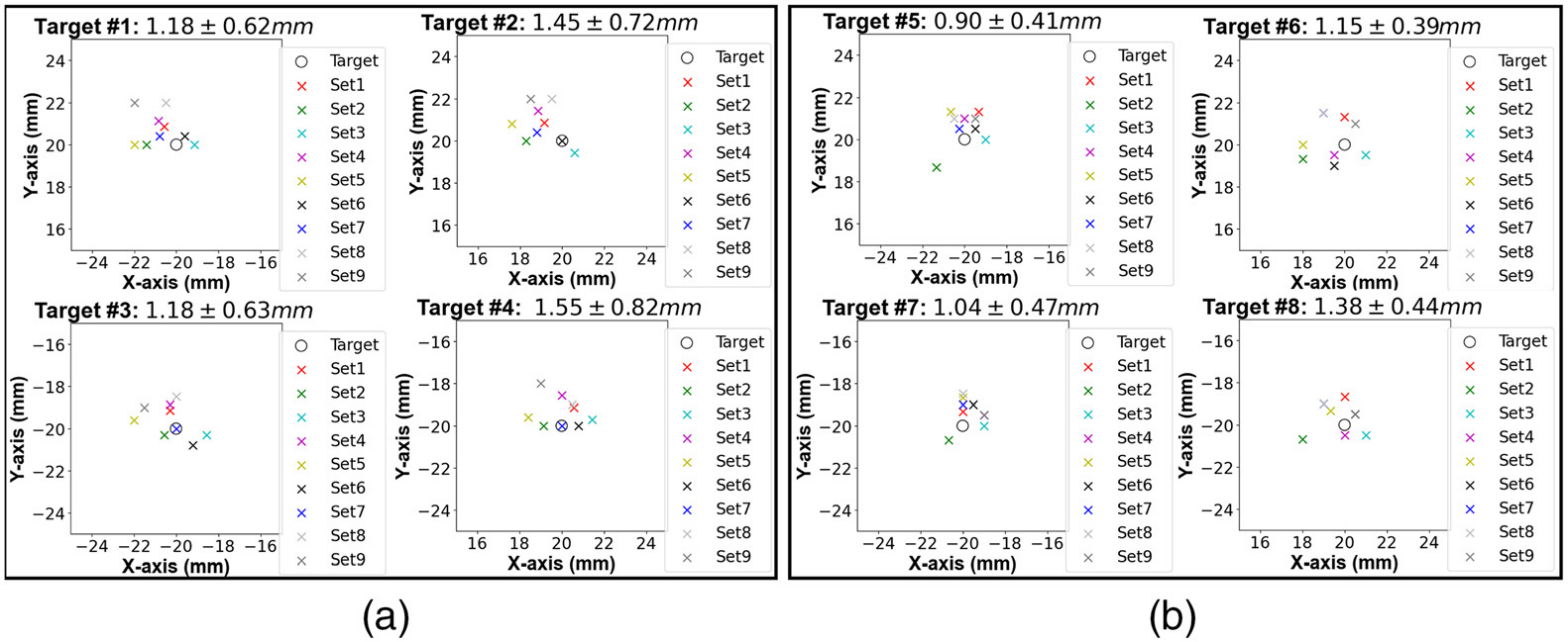
Visualization of target overlay results. Scatter plot of the overlay error at each target position located at (a) 5 cm and (b) 10 cm from the base. The overall target overlay error was 1.29±0.57  mm.

The overall image overlay error over 56 targets (8×9) was 1.29±0.57  mm. The back projection of nine trials for each individual target is shown in Fig. 6. The Shapiro–Wilk tests result did not show a significant departure from the normality for each of eight positions and there were no statistically significant differences between each individual target overlay error (one-way ANOVA, F(2,8)=0.81, p=0.58).

The visual needle targeting error over 24 trials on eight targets was 2.13±0.52  mm.

### 3.2 Experiment 2: Performance of HoloLens AR Guidance in Needle Placement Task

**Fig. 7:**
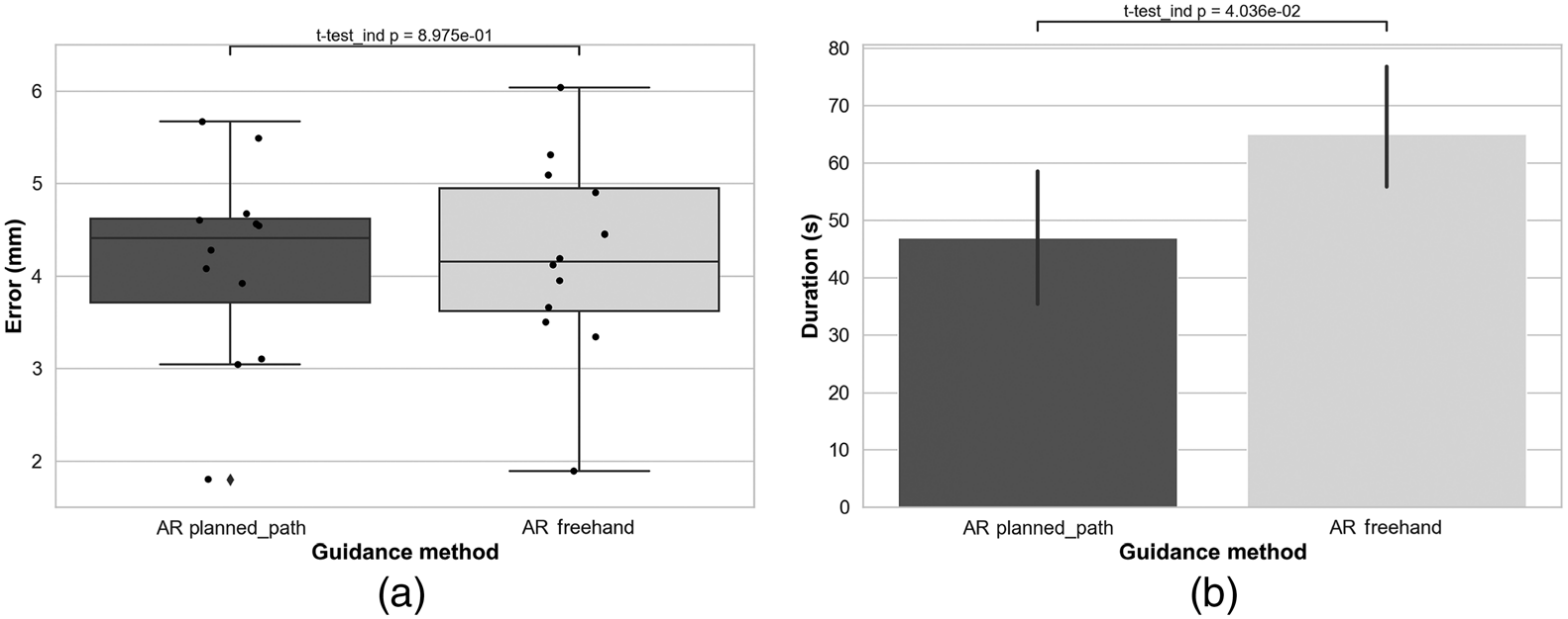
Results of needle placement performance. (a) Comparative box-and-whisker plot of the needle placement error for the AR guided planned-path and free-hand method. Each dot represents a measurement of error for each insertion. (b) Comparative bar graph of the needle placement time for the AR guided planned-path and free-hand method.

The needle placement error with AR-guided planned path was 4.14±1.08  mm and was normality distributed (p=0.425), whereas the needle placement error with AR-guided free-hand was 4.20±1.08  mm and was normality distributed (p=0.955). They were similar [Student’s t-test, p=0.90, Fig. 7(a)]. The needle placement time with the AR-guided planned path and with the AR-guided freehand method was 47±22  s and 65±19  s, respectively [Mann–Whitney U test, p=0.04, Fig. 7(b)].

Operators commented that when using the AR-guided planned-path method, the digital needle plan was occasionally not perfectly aligned with the actual needle, although the projection consistently appeared parallel to the needle. Operators also reported when using the AR-guided free-hand method, there was a noticeable lag before the digital needle realigned properly when the tracked needle was moving, and the displayed digital needle overlay occasionally was not stable visually due to this display or system flicker.

### 3.3 Experiment 3: Feasibility of HoloLens Guidance in Free-Hand TP Seed Implantation Procedure

Of the total five implantations, two tissue markers were successfully implanted within seminal vesicles, and one marker was successfully implanted within a pseudo lesion [Figs. 5(f) and 5(h)]. The other two tissue markers were placed beyond the intended pseudo targets; one was placed 0.9 mm [Figs. 5(e) and 5(g)] from the target lesion boundary and the other was placed 7 mm from the target lesion boundary.

## 4 Discussion

In this study, we presented a novel HoloLens AR navigation system for transperineal prostate (TP) biopsy. The AR navigation system superimposed the planned needle path and monitored progress of the needle by continuously tracking image patterns that were rigidly attached to the patient and the device.

The HoloLens AR system had excellent accuracy and minimal visual needle targeting error over the volume of the prostate. To objectively evaluate the HoloLens AR, interpupillary distance was calibrated and the camera was placed at the optimal ocular position (center and parallel to the lens display). The HoloLens AR system accuracy was not reported using typical target registration error (TRE). TRE should be measured in 3D space with the 3D display output of HoloLens AR, which requires stereoscopic vision and involves user-based perception. Although the current measurement was conducted on the monocular display, it incorporates information from binocular vision under the assumption of optimal convergence and accommodation of the left and right lenses.

The performance of AR-guided TP needle navigation was evaluated by comparing the AR planned path and the AR free-hand method. We found that operators using HoloLens AR guidance with both methods were able to place needles accurately and consistently. With the planned path method, as long as the image pattern on patient reference frame was within the line of sight of the HoloLens’ camera, the planned needle trajectory was continuously visible. But under these conditions the needle entry site on the perineum needed to be initialized prior to the procedure. On the other hand, the free-hand method provided the user with the flexibility of choosing the needle entry site and provided real-time visualization of the progress of the needle during insertion. However, for this technique, a needle reference frame was required on the needle shaft or hub, which has ergonomic and sterility disadvantages. To display both the virtual needle and the anatomic target simultaneously, both image patterns on the patient reference frame and the needle reference frame needed to be kept within the line of sight of the HoloLens’ camera.

In the feasibility experiment with seed implantation in a pelvic phantom, pseudo lesions were used to mimic prostate cancer. The pseudolesions were artificially added to the preprocedural MRI and were transformed to the postinterventional MRI based on seven fiducial markers on the patient reference frame for postseed implantation position evaluation. The discrepancy in relative position of fiducial markers and the pelvic phantom between two scans and residual error associated with transformation could contribute to the measurement error. A phantom with MRI visible lesions would be ideal and eliminate the introduced measurement error. We also observed that during the release of the seed, it required both hands to operate and the user tended to advance the needle unintentionally. This may explain why the implanted seed tended to be slightly deep to the target, especially for the first trial with a result of 7 mm too deep.

The expected tolerable error in prostate biopsies was estimated to be 5 mm, which is reminiscent of the smallest detectable target in prostate imaging (3.0 T MRI).^20^ A $0.5\;{\mathrm{cm}}^{3}$ prostate cancer volume has been proposed as the limit of clinically significant prostate cancer foci volume^21^ and is equivalent to a sphere with a radius of 5 mm. The needle placement error with HoloLens AR guidance was just above 4  mm±1  mm. The average error is near the expected tolerable error and is in the range of potential clinical relevance, depending on the need for accuracy. Other systems for improved accuracy of TP biopsies include a grid template with a 3 mm target distance error in a phantom study^22^ and a robotics with a 2 mm error in a phantom study.^23^^,^^24^ However, our HoloLens AR system only provides visual guidance, without any hardware or physical constraints. Conventional manual guidance with US imaging is subject also to volume averaging of the 2D US slice, which may also be in the 3 mm range. Manual needle placement may veer off target even with a rigid grid, especially with a beveled needle in a stiff prostate tissue. Thus, this AR approach with visual cues may compare favorably to the cumulative error range of the manual approach (without robotics or automation hardware).

The needle placement error with the AR-guided system was a combination of the system error and subjective error introduced by human factors (operators). The system error was 1.29±0.57  mm in image overlay error and 2.13±0.52  mm in visual needle targeting error, well within the expected tolerable error. Operators’ subjective error was caused by various factors including the technique of inserting a needle and the depth decision of when the target was reached. Moreover, the placement accuracy also could be affected by the operators’ experience with the HoloLens. This includes such factors as consistent viewing angles and fast adjustment to the simultaneous visualization of digital and physical objects at different focal distances. The needle placement accuracy of using AR-guided system could be improved via more operator training. Each operator in this study was trained for ∼20 to 30 min with the HoloLens prior to the experiment. More time and experience with a HoloLens system could improve the performance, but definition requires further investigation.

HoloLens AR guidance offered the advantage of a direct line of sight through the lens display with an intuitive and hands-free user experience. HoloLens AR required user-dependent calibration and adjustment.^18^ Accurate superimposition of digital objects is highly dependent on the position of a user’s eyes relative to the lens and the user-based adjustment and fit of the device. A small parallel shift in needle alignment was observed especially when the users moved their head back to the phantom after looking away for some time. This was due to the alteration of the viewing angle through the lens. To ensure precise and comfortable visualization during procedures, users should be well-trained regarding how to properly wear, adjust, and use the HoloLens. With the current device and tracking Vuforia SDK performance, repositioning lag (about 0.3 s), and augmentation jitter occurred when tracking a moving object such as the needle. Further improvement of technology hardware or software could reduce the lag and jitter thereby enhancing the user experience.

A limitation of the presented transperineal AR guidance system is that it is based on preprocedural 3D volumetric images and therefore cannot make automatic real-time corrections for organ motion and deformation without an additional US. Likewise, the patient positioning during the preprocedural imaging may also impact organ and target location, as the MRI is supine and the biopsy most often lithotomy position. One possible solution may be to use real-time US fused to preprocedural 3D volumetric images^25^ to track lesion position during needle insertion. When combined with real-time US, real-time segmentation^26^ with subsequent deformable registration^27^ is now computationally feasible with advanced processors. Although speculative, this AR system might directly address unmet clinical needs such as depth of needle insertion during out of rectum TPUS guided TP needle procedures, when the needle visualization has reduced conspicuity.

## 5 Conclusion

A HoloLens-based AR system was developed to facilitate and assist needle trajectory guidance for needle-based transperineal procedures. The results of this study suggest that the HoloLens AR can provide an accurate image overlay for digital object visualization and reliable guidance for free-hand transperineal procedures, potentially with TRUS or TPUS guidance. The HoloLens displays a true 3D hologram of the digital object in a 3D environment, enabling a more user-friendly immersive experience as well as increased awareness of the real physical environment during the procedure. Such a system may address current shortcomings in standardized approaches to free-hand TP biopsy and ablation. Future refinements to the AR system could define clinical value, especially as needles (and possibly US) procedures move from the rectum to the perineum.
